# National Survey of Radiation Dose and Image Quality in Adult CT Head Scans in Taiwan

**DOI:** 10.1371/journal.pone.0131243

**Published:** 2015-06-30

**Authors:** Chung-Jung Lin, Greta S. P. Mok, Mang-Fen Tsai, Wei-Ta Tsai, Bang-Hung Yang, Chun-Yuan Tu, Tung-Hsin Wu

**Affiliations:** 1 School of Medicine, National Yang-Ming University, Taipei, Taiwan; 2 Department of Radiology, Taipei Veterans General Hospital, Taipei, Taiwan; 3 Biomedical Imaging Laboratory, Department of Electrical and Computer Engineering, Faculty of Science and Technology, University of Macau, Macau, China; 4 Department of Biomedical Imaging and Radiological Sciences, National Yang Ming University, Taipei, Taiwan; 5 Department of Radiation Oncology, Tzu Chi General Hospital Dalin Branch, Chiayi, Taiwan; 6 Department of Nuclear Medicine, Taipei Veterans General Hospital, Taipei, Taiwan; 7 Department of Radiology, Mackay Memorial Hospital, Taipei, Taiwan; 8 Association of Medical Radiation Technologists, Taipei, Taiwan; University of Nebraska Medical Center, UNITED STATES

## Abstract

**Introduction:**

The purpose of the present study was to evaluate the influence of different variables on radiation dose and image quality based on a national database.

**Materials and Methods:**

Taiwan’s Ministry of Health and Welfare requested all radiology departments to complete a questionnaire for each of their CT scanners. Information gathered included all scanning parameters for CT head scans. For the present analysis, CT machines were divided into three subgroups: single slice CT (Group A); multi-detector CT (MDCT) with 2-64 slices (Group B); and MDCT with more than 64 slices (Group C). Correlations between computed tomography dose index (CTDI) and signal-to-noise ratio (SNR) with cumulated tube rotation number (CTW(n)) and cumulated tube rotation time (CTW(s)), and sub group analyses of CTDI and SNR across the three groups were performed.

**Results:**

CTDI values demonstrated a weak correlation (r = 0.33) with CTW(n) in Group A. SNR values demonstrated a weak negative correlation (r = -0.46) with CTW(n) in Group C. MDCT with higher slice numbers used more tube potential resulting in higher effective doses. There were both significantly lower CTDI and SNR values in helical mode than in axial mode in Group B, but not Group C.

**Conclusion:**

CTW(n) and CTW(s) did not influence radiation output. Helical mode is more often used in MDCT and results in both lower CTDI and SNR compared to axial mode in MDCT with less than 64 slices.

## Introduction

Increased use of CT in daily practice made its associated radiation dose a global medical concern [1–4]. Identifying excessive radiation dose is not easy and some epidemiological studies have indicated that CT radiation doses are large enough to increase cancer risk [5–7]. Several national studies have provided diagnostic reference levels for different CT protocols, making international comparisons feasible [8–13]. For example, national surveys in Germany and the UK demonstrated higher radiation doses in 4-slice CT than in single slice CT [14, 15]. However, many of these studies were performed between the late 1990s and early 2000s, i.e., before the widespread introduction of multi-detector (MDCT). The use of more detectors raises the radiation dose, and later national surveys with higher percentages of MDCT scans found higher dose reference levels (DRLs), i.e., value of the third quartile (75%) of the overall population, than those found in the earlier studies [8, 16, 17].

Although the increased radiation dose with MDCT has been attributed to over-beaming [18, 19], factors such as detector design, scanning modes, tube voltage and tube current also influence the dose. Strategies have been made to reduce radiation by alternating scanning parameters. Nevertheless, its influence on actual radiation output from MDCT on a national scale remains unclear. A second concern is that whether the use of older CT machines results in higher radiation doses and lower image quality. Since the scan length is relatively constant and the geometry is fairly rigid for the adult head as compared to other body parts [9, 18], it is the ideal model for evaluating radiation doses from different CT scanners.

To better understand how the image quality intervened with radiation doses for different CT scanners currently used in Taiwan, Taiwan’s Ministry of Health and Welfare funded this project in cooperation with the National Association of Radiological Technicians. Through an analysis of the results from this nationwide questionnaire, we aimed to establish our DRLs and to answer the following questions: 1) Will the previous workload of the tube influence radiation dose and signal-to-noise ratio (SNR) in head CT scans? 2) Will CT with more detectors lead to a higher radiation dose as compared to CT with fewer detectors?

## Materials and Methods

### Questionnaire design and distribution

Between January and December 2012, Taiwan’s Ministry of Health and Welfare (MOHW) and the Association of Medical Radiation Technologists in Taiwan conducted a nationwide investigation of CT radiation dosage and image quality. The 242 CT scanners in service and registered in a national database were enrolled for this study. There are currently no CT scanners in private practice in Taiwan. The study was approved by the Mackay Memorial Hospital Institutional Review Board on Mar. 24, 2012 and valid till Mar. 23, 2013. (period of Carry Out Approved Activities: Mar. 24,2012~ Dec. 10, 2012) the constitution and operation of this review board are according to the guidelines of ICH-GCP, the patient records/information was anonymized and de-identified prior to analysis. One questionnaire was collected for each CT scanner in every hospital in Taiwan, prepared by the on-site technicians. The first part of the questionnaire sought basic information about the CT scanner, including the vendor, number of detectors. Previous workload indicators, i.e., cumulated tube rotation number (CTW(n)) and cumulated tube rotation time (CTW(s)) were also collected from the console display. In this study, CTW(n) is defined as the cumulated number of X-ray tube rotation since the installation of the scanner when the questionnaire was collected, while CTW(s) is defined as cumulated tube rotation time (in seconds) since the installation of the scanner. The age of the scanner was excluded since it cannot accurately reflect the previous workload of the X-ray tube. For the second part of the questionnaire, the on-site technician was asked to retrospectively choose ten routine adult head CT scans from the respective CT consoles, and to report the following scanning parameters: milliampere-second (mAs), kilovoltage (kV), slice thickness, pitch, reconstruction kernel, computed tomography dose index (CTDI), and dose length product (DLP). None of the CT scans used iterative reconstruction when the survey took place. Although tube modulation effectively reduces radiation dose by 22%-50%, scans using tube current modulation were excluded from the current study due to their fairly small sample size (n = 112, 5.8%) [20].

**Fig 1:**
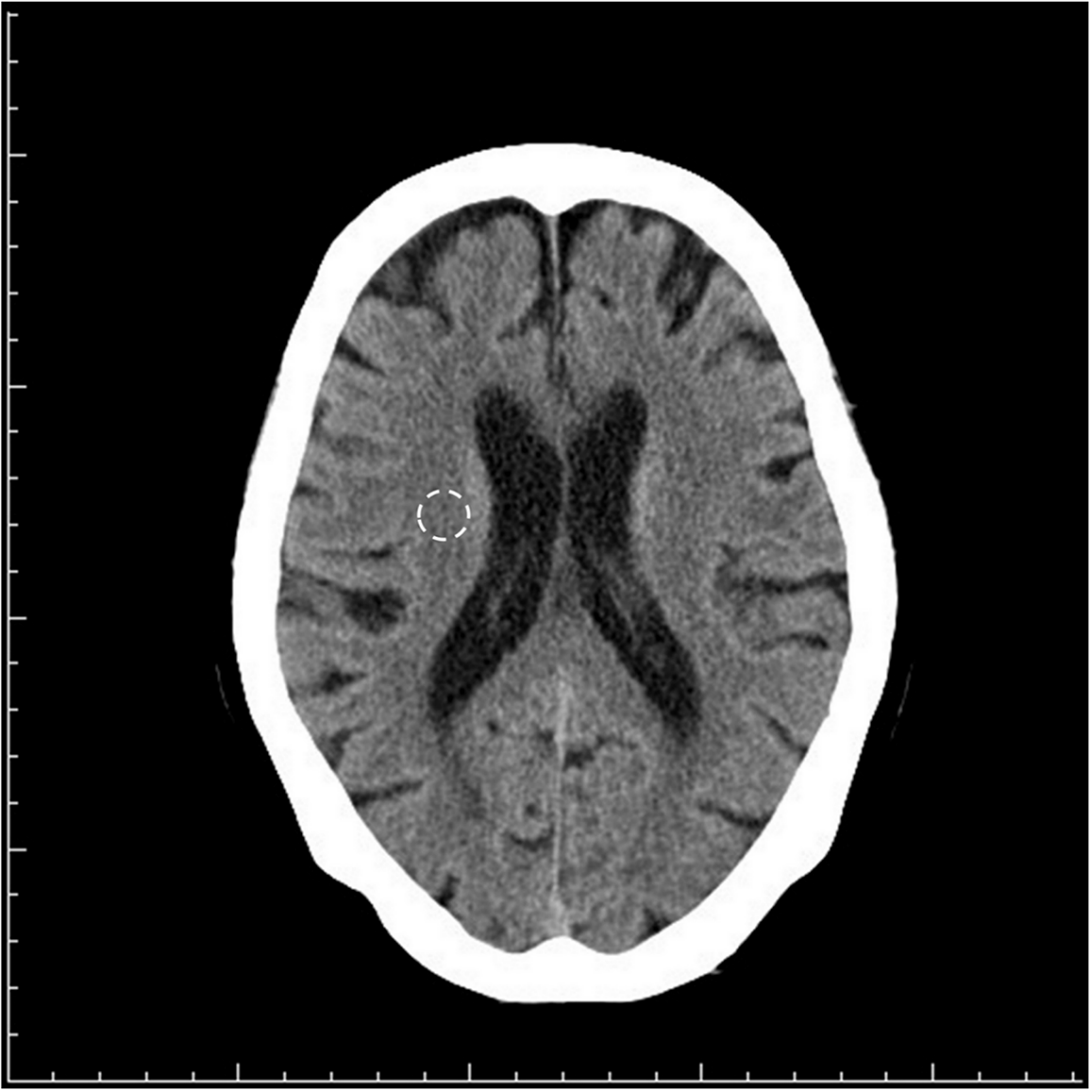
The ROI chosen for calculating the SNR.

Effective dose was calculated using DLP-to-effective dose conversion factor, 0.0021 mSv/mGy-cm for adult head scan, which was derived based on the definition of effective dose defined in ICRP Publication 103 [21]. The CTDI was retrieved from the dose information page of the console. For single slice CT without a dose information page, the CTDI was calculated using ImPACT (Version 1.0.4, Imaging Performance Assessment of CT Scanners, London UK) based on the acquisition parameters. The image quality for each CT scan was assessed based on a manually drawn region-of-interest (ROI) of 100 mm^2^ located in the right periventricular was assessed based on a manually drawn ROI of 100 mm^2^ located in the right periventricular brain parenchyma, immediately next to the right ventricular wall (Fig 1). If there were old infarcts or other insults, we moved the ROI anteriorly-posteriorly along the right ventricular wall to locate a normal brain region. The SNR was defined as ROI _mean_/ROI _standard deviation_. We collected the questionnaires from the technicians for data anonymization and further analysis.

### Data validation and analysis

**Fig 2:**
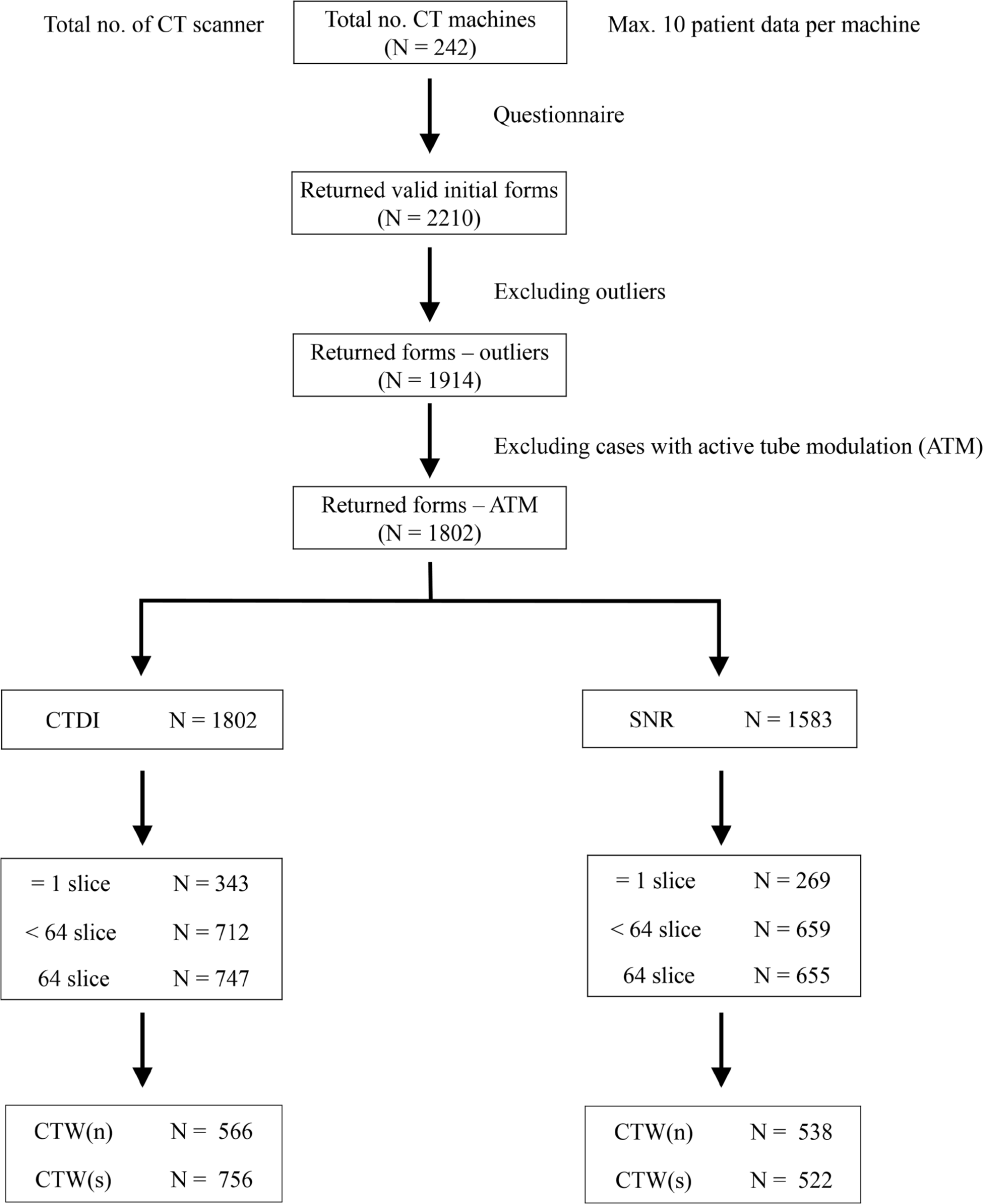
The work flow diagram demonstrates how the raw data from the questionnaire was rearranged into the final dataset.

For each questionnaire, missing values were left blank while other available data were still used. The questionnaire was considered invalid if values of both CTDI and SNR were missing. Entry values of > 1.5 times the inter-quartile range from the mean were excluded from subsequent analysis. Given the fundamental differences in the hardware design of single slice CT and MDCT, all CT scanners were divided into 3 subgroups: single slice CT(Group A); MDCTs with a detector number <64 (Group B); MDCTs with ≥64 detectors. Subgroup comparisons of CTDI and SNR were also done for different scan modes and between Group B and C. The workflow of the study was summarized in Fig 2.

### Statistics

The relationship of CT hardware parameters (CTW(n) and CTW(s)) with CTDI and SNR was assessed using Pearson correlations for all three groups. The differences of characteristics as well as CTDI and SNR among the three groups were tested by ANOVA with Bonferroni adjustment for continuous variables and chi-square for categorical variables. Subgroup analysis comparing helical and axial scanning modes were performed between Group B and C using *t*-tests. All statistical analyses were performed using MedCalc (Version 13.2.2, MedCalc Software, Ostend, Belgium).

## Results

The geographic distribution of the surveyed CT scanners is shown in **Table 1**. The overall questionnaire response rate was 81.6%. There were significantly less returned valid data for Group A than for the other two groups in all areas except the east area. There were no valid data for Group C in the east area because all the scans in this category were performed with active tube modulation and were thus excluded. There were similar proportions of each of the three groups in the north, middle and south areas.

**Table 1:** Geographic Distribution of CT Scanners and Valid Questionnaires.

	Group A (Single slice)	Group B (< 64 slice)	Group C (≥ 64slice)
Total questionnaires sent (N = 2210)	475	851	884
Total number of valid questionnaires (N = 1802)	343 (72.2%)*	712 (83.7%)*	747 (84.5%)*
North area	105 (11.9%) ‡	390 (44.2%) ‡	388 (43.9%) ‡
Central area	73 (20.8%) ‡	118 (33.6%) ‡	160 (45.6%) ‡
South area	116 (22.8%) ‡	194 (38.1%) ‡	199 (39.1%) ‡
East area	49 (80.3%) ‡	10 (19.6%) ‡	0
All areas	343 (19.3%) ‡	712 (40.2%) ‡	747 (42.1%) ‡

*Percentage of valid returned questionnaires to total number of questionnaires sent.

‡ Percentage of the valid questionnaires of each group to the total questionnaires in the specific area (row percentages sum to 100%).

The technical characteristics and dose information of CT scanners in all groups are shown in **Table 2**. Group A had the smallest sample size, lowest CTDI (45.73 ± 16.04 mGy) and lowest CTW(n) (2.03×10^5^±2.60×10^5^ times) values. The CTW(n) was significantly higher in Group C (4.46×10^5^± 3.64×10^5^ times) than in Group B (2.71×10^5^±2.43×10^5^ times). The effective dose was lowest in Group A (1.00 ±0.64 mSv), followed by Group B (1.75 ±0.61 mSv) and Group C (1.99 ±0.58 mSv). The DRLs were significantly lower in Group A than in the other two groups.

**Table 2:** Technical Characteristics and Radiation Doses Information for the CT scanners in Three Groups.

	Group A (Single slice)	Group B(< 64 slice)	Group C (≥ 64slice)
Number	343*	712	747
CTW(n) (times)	2.03×10^5^±2.60x10^5^	2.71×10^5^±2.43×10^5^ ‡	4.46×10^5^±3.64×10^5^ ‡
CTW(s) (seconds)	N/A	2.17×10^5^±7±1.90×10^5^	2.00×10^5^±1.38×10^5^
CTDI (mGy)	45.73±16.04*	59.31±12.08	58.05±11.78
Effective dose (mSv)	1.00±0.64‡	1.75±0.61‡	1.99±0.58‡
DRL of CTDI (mGy)	57.13	68.31	65.11
DRL of DLP (mGy-cm)	691.2	1021.69	1118.31
Vendor (N)(G/S/P/T/O)^▲^	177/30/10/71/99	360/160/60/133/30	171/125/190/279

*Significantly different from the other two groups.

‡Significantly different from all other groups.

^▲^G:GE; S:Siemens; P:Philips; T:Toshiba; O: Other vendors.

The acquisition parameters are summarized in **Table 3**. The tube current was lowest in Group A, followed by Group B and Group C. However, the duration of active tube current was longest in Group A, followed by Group B and Group C. The lowest tube current time product was in Group A (263.87±81.98 mAs), followed by Group B (280.23±65.19 mAs) and Group C (302.81±97.40). The tube voltage of Group C (120.17±3.43 kV) was significantly lower than other two groups, but the difference was < 1 kV. The pitch in Group C was higher (1.03±1.29) than in the other two groups. Axial mode was more often used in Group B (84.4%) than in Group C (59.7%). The SNR in Group A (8.50±1.93) was highest, followed by Group B (7.45±1.72) and Group C (7.09±1.69). The slice thickness in Group A (7.6±2.4 mm) was thicker than in the other two Groups. There were no significant difference in slice thickness between Group B and Group C.

**Table 3:** Acquisition Parameters of CT Head Scans for the Three CT Groups.

	Group A (Single slice)	Group B (< 64 slice)	Group C (≥ 64 slice)
Number	289	681	715
Tube current (mA)	164.49±58.45‡	242.61±74.63‡	294.45±90.41‡
Duration of active tube current (s)	1.91±1.143‡	1.27±0.62‡	1.07±0.53‡
Tube current time product (mAs)	263.87±81.98	280.23±65.19	302.81±97.40*
Tube voltage (kV)	120.95±3.21	121.06±4.90	120.17±3.43*
Pitch	1	0.88±0.62	1.03±1.29*
Helical mode (%)	0‡	111 (15.6%)‡	301 (40.3%)‡
Slice thickness (mm)	7.62±2.36*	4.83±1.37	4.593±1.28
CTDI (mGy)	45.73±16.04*	59.31±12.08	58.05±11.78
SNR	8.50±1.50±1.93‡	7.45±1.72‡	7.09±1.69‡

*Significantly different from the other two groups.

‡Significantly different from all other groups.

**Fig 3:**
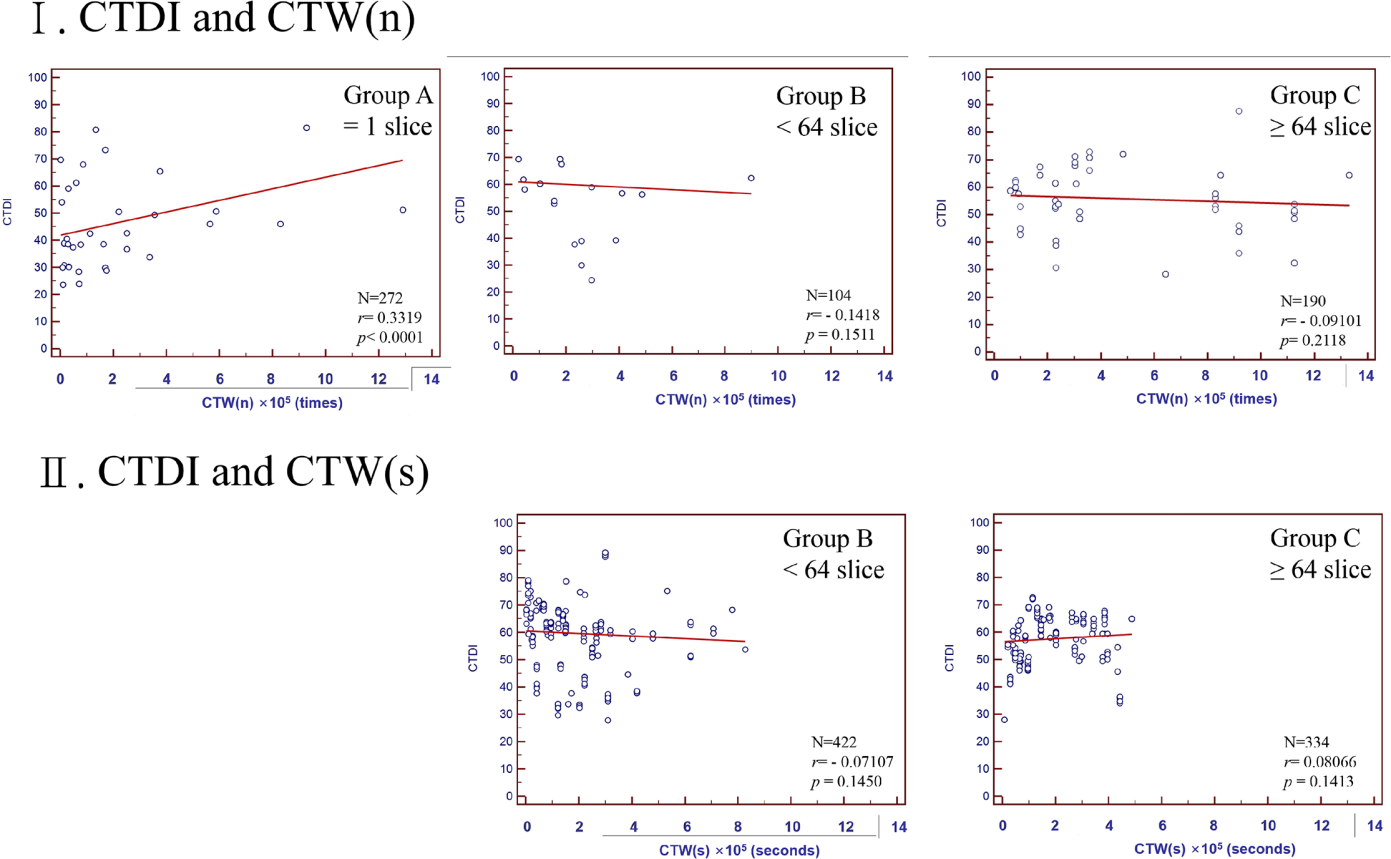
Correlation of CTDI with CTW(n) and CTW(s) for Group A = 1 slice, Group B <64 slices, and Group C≥64 slices.

**Fig 4:**
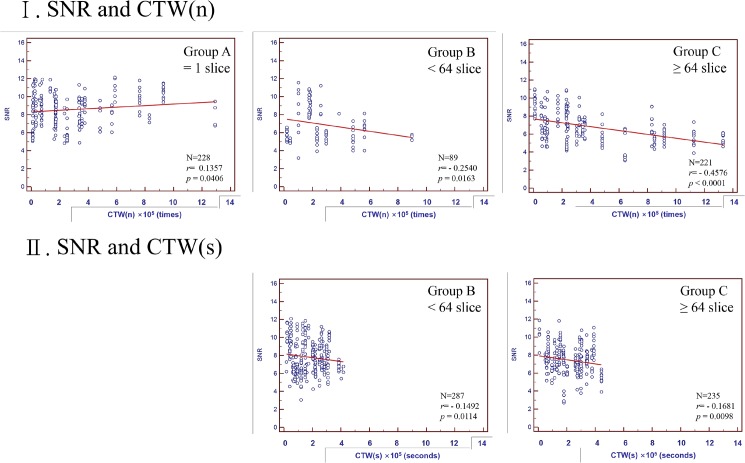
Correlation of SNR with CTW(n) and CTW(s) for Group A = 1 slice, Group B <64 slices, and Group C≥64 slices.

There were no correlations between CTDI and CTW(n) or CTW(s) (Fig 3) in all groups, except that CTDI demonstrated a weak correlation (r = 0.33) with CTW(n) in Group A. There were no correlations between SNR and CTW(n) or CTW(s) (Fig 4) in all groups except that SNR demonstrated a weak negative correlation (r = -0.46) with CTW(n) in Group C.

**Fig 5:**
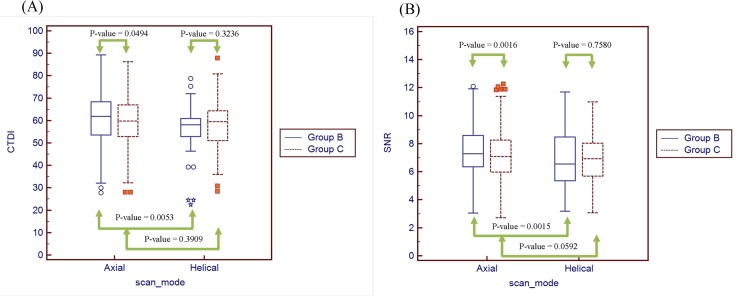
Influence of different scan modes and detector numbers (Group B<64 slices, and Group C≥64 slices) on (A) CTDI and (B) SNR.

The subgroup analysis of Group B and C is demonstrated in Fig 5. Results showed significantly higher CTDI in Group B than in Group C (59.92±10.77 vs. 58.98±11.54 mGy, *p* = 0.0494) in axial mode while not in helical mode (57.81±9.77 vs. 58.5±9.98 mGy, *p* = 0.3236). Findings were consistent in subgroup comparisons of SNR: SNRs were significantly higher in Group B than in Group C in axial mode (7.55±1.62 vs. 7.20±1.67, *p* = 0.0016) but not in helical mode (7.01±2.03 vs. 6.95±1.70, *p* = 0.7580). In Group B, the use of axial mode resulted in higher CTDI (59.92±10.77 mGy) and SNR (7.55±1.62) values than those in the use of helical mode. However, there were no significant differences in CTDI or SNR between the two scanning modes for Group C.

## Discussion

The number of MDCTs installed is correlated with the economic development of the individual areas. Given the lower radiation dose in single slice CT, patients are likely to receive 13%-50% less radiation exposure if the head scan is performed in the east area since the majority of the CT scanners there is single slice CT. This information can be served as a geographic baseline and be used to optimize the epidemic research in medical radiation exposure. Our study found no strong evidence that more used X-ray tubes would lead to higher CTDI or inferior SNR. The concern about older CT scanners would have inferior performance seems to be ungrounded. Regular maintenance and quality control appears to be sufficient to ensure adequate radiation dose and image quality for daily CT head scans.

The overall DRL of DLP in Taiwan was 999 mGy-cm, lower than those found in Japan, Korea, Kenya, Syria and the U.K. (**Table 4**) [22]. However, the DRLs in Group B and Group C were all > 1000 mGy-cm, very close to the values in Korea and Japan. Our study confirms that with the introduction of MDCT, the associate CTDI increased. However, MDCT with more detectors (Group C) did not necessarily lead to significantly higher CTDI than MDCT with fewer detectors (Group B). Given the very similar kV, we hypothesize that the higher tube current combined with longer pitch in Group C resulted in similar CTDI for the two groups. However, Group C displayed higher effective doses than Group B by 14%, probably due to wider scan ranges used in this group.

**Table 4:** Comparison of International DRLs of DLP for Adult CT Head Scans.

	Number of scans	Range of detector numbers in all CT	DRL (mGy-cm)	Order
U.K (1999)	N/A	N/A	1050	4
Korea (2008)	128764	N/A	1056	3
Syria (2009)	N/A	N/A	900	5
Switzerland (2010)	187	1–256	1000	8
Malta (2012)	94	2–64	736	9
Kenya (2012)	84	1–64	1575	1
Japan(2012)	4587	1–320	1120	2
Ireland (2012)	7778	2–128	940	7
Taiwan (2012)	1802	1–256	999	6

All the average slice thicknesses from MDCT were < 5 mm, which is the minimal requirement in the ACR-ASNR guidelines [23]. With the introduction of MDCT, the use of multi-planar reformation has become increasingly popular. Thus, the slice thickness in MDCT becomes thinner than that of the single slice CT. To compensate for the reduced SNR in a thinner slice, MDCT enhances the tube voltage, however, the overall SNR decreased as the number of detector increased. Group A demonstrated the highest SNR (8.50±1.93) mainly due to thicker slice (7.62±2.36 mm) used, given that it had lower tube voltage than the other groups.

Subgroup analysis found lower SNR values for Group C than for Group B, particularly in axial mode. Given similar slice thickness and CTDI, the remaining attributing factors are pitch and reconstruction kernel. Helical mode reduces unwanted motion artifact at the cost of lowered SNR and higher CTDI [17] and is conventionally reserved for patients with, for example, disturbed consciousness or head trauma. When using CT scanners with more detector numbers in combination with multiplanar reformation, technicians often prefer to use helical mode in head scans instead of axial scans to reduce patient positioning time, which may lead to increased radiation dose between the two modes.

This is the first establishment of national DRL. The initial results showed that our DRL was placed 6^th^ among 10 countries. All the examinations with reported values exceeding the DRL were identified and the corresponding chief radiologic technicians would receive a notification by the association. Reduction of radiation doses was advised and follow-up of the optimization would be performed by the association in a 6–12 months interval. The national optimization process is still ongoing and therefore the update is not clear yet.

There are a few limitations in our study. First of all, this national survey was not obligatory and the response rate was <100%. The lower response rate in Group A may indicate that the study might not be fully representative of the current performance of single slice CT. Second, since it was a national investigation, user-adjustable variables such as mAs, kV and slice thickness, were not homogenous across three groups, which made further analysis of the individual impact of each aforementioned parameter on effective dose and image quality challenging. Finally, tube current modulation and iterative reconstruction in CT serve to reduce radiation dose [18, 20, 24]. However, most neuroradiologists in Taiwan do not appreciate the waxy appearance of current iterative reconstruction [24]. Besides, the suboptimal low contrast-to-noise ratio in iterative reconstruction remains a major concern in the head CT scans [24–26]. Therefore, iterative reconstruction is still in its preliminary phase in Taiwan. As for the tube modulation technique, Smith *et al*. found 30–50% dose reduction when comparing 64-slice CT with tube modulation with 16-slice CT without modulation. However, it did not reduce much radiation dose (<10%) for the same CT scanner. Meanwhile some artifact appears near the vertex of head, and thus this technique was not commonly applied in head CT scans in the majority of the hospitals [27]. Due to the fairly small sample size, we excluded the associate iterative reconstruction and tube current modulation data to facilitate the subsequent statistical analysis. Nevertheless, the feasibility and robustness of these techniques might change as they continue to advance. Their dose reducing effects and actual influence on image quality warrant further evaluation in a national level study.

In general, MDCT radiation doses increased along with increased detector numbers due to over-beaming [14]. In contrast, the SNR decreased significantly along with increased detector numbers due to the thinner slice thickness from MDCT, as the “acquire thin and view thick” strategy has been widely adapted in current practice [18]. Other possible factors such as hardware design, reconstruction kernels or filters may also influence radiation dose and image quality but analysis of these factors was beyond the scope of this study.

## Conclusion

The previous workload of the tube was not correlated with CTDI or SNR. MDCT with < 64 slices demonstrated higher CTDI and SNR than those with > 64 slices in both axial and helical mode. Helical mode was used more often than axial mode in MDCT with more slices in current practice, and resulted in lower SNR. Optimizing the scanner parameters and reconstruction kernel is recommended to provide diagnostic imaging of CT head scan with a reasonable radiation dose. Retrospective dose calculations using the scanner-specific CTDIw and its variation across the measurements for the same scanner are also warranted for the future study.

## References

[1] Kalra MK, Maher MM, Toth TL, Hamberg LM, Blake MA, Shepard JA, et al Strategies for CT radiation dose optimization. Radiology. 2004 3; 230(3):619–28. . 14739312 10.1148/radiol.2303021726

[2] Amis ES Jr, Butler PF, Applegate KE, Birnbaum SB, Brateman LF, Hevezi JM, et al American College of Radiology white paper on radiation dose in medicine. J Am Coll Radiol. 2007 5; 4(5):272–84. . 17467608 10.1016/j.jacr.2007.03.002

[3] Wintermark M, Lev MH. FDA investigates the safety of brain perfusion CT. Am J Neuroradiol. 2010 1; 31(1):2–3. 10.3174/ajnr.A1967 19892810 PMC7964089

[4] Trattner S, Pearson GD, Chin C, Cody DD, Gupta R, Hess CP, et al Standardization and optimization of CT protocols to achieve low dose. J Am Coll Radiol. 2014 3; 11(3):271–8. 10.1016/j.jacr.2013.10.016 24589403 PMC3969855

[5] Pearce MS, Salotti JA, Little MP, McHugh K, Lee C, Kim KP, et al Radiation exposure from CT scans in childhood and subsequent risk of leukaemia and brain tumours: a retrospective cohort study. Lancet. 2012 8 4; 380(9840):499–505. 10.1016/S0140-6736(12)60815-0 . 22681860 PMC3418594

[6] Mathews JD, Forsythe AV, Brady Z, Butler MW, Goergen SK, Byrnes GB, et al Cancer risk in 680,000 people exposed to computed tomography scans in childhood or adolescence: data linkage study of 11 million Australians. Br Med J. 2013 5 21; 346:f2360 10.1136/bmj.f2360 . 23694687 PMC3660619

[7] John D, Boice Jr. The Boice Report #14. Nation Council on Radiation Protection and Measurements. 2013 July. Available: http://ncrponline.org/PDFs/BOICE-HPnews/14_UNSCEAR_Vienna_July2013.

[8] Foley SJ, McEntee MF, Rainford LA. Establishment of CT diagnostic reference levels in Ireland. Brit J Radiol. 2012 10; 85(1014978):1390–7. 10.1259/bjr/15839549 . 22595497 PMC3474022

[9] Fukushima Y, Tsushima Y, Takei H, Taketomi-Takahashi A, Otake H, Endo K. Diagnostic reference level of computed tomography (CT) in japan.Radiat Prot Dosimetry. 2012 8; 151(1):51–7. 10.1093/rpd/ncr441 . 22147925

[10] Korir GK, Wambani JS, Korir IK. Patient doses using multidetector computed tomography scanners in Kenya.Radiat Prot Dosimetry. 2012 8; 151(2):267–71. 10.1093/rpd/ncr484 . 22279198

[11] Choi J, Cha S, Lee K, Shin D, Kang J, Kim Y, et al The development of a guidance level for patient dose for CT examinations in Korea. Radiat Prot Dosimetry. 2010 2; 138(2):137–43. 10.1093/rpd/ncp236 . 19864327

[12] Kharita MH, Khazzam S. Survey of patient dose in computed tomography in Syria 2009. Radiat Prot Dosimetry. 2010 9; 141(2):149–61. 10.1093/rpd/ncq155 . 20511400

[13] Treier R, Aroua A, Verdun FR, Samara E, Stuessi A, Trueb PR. Patient doses in CT examinations in Switzerland: implementation of national diagnostic reference levels. Radiat Prot Dosimetry. 2010 12; 142(2–4):244–54. 10.1093/rpd/ncq279 . 20926508

[14] Brix G, Nagel HD, Stamm G, Veit R, Lechel U, Griebel J, et al Radiation exposure in multi-slice versus single-slice spiral CT: results of a nationwide survey. Eur Radiol. 2003 8; 13(8):1979–91. . 12687286 10.1007/s00330-003-1883-y

[15] Shrimpton PC, Hillier MC, Lewis MA, Dunn M. National survey of doses from CT in the UK: 2003. Brit J Radiol. 2006 12; 79(948):968–80. . 17213302 10.1259/bjr/93277434

[16] Palorini F, Origgi D, Granata C, Matranga D, Salerno S. Adult exposures from MDCT including multiphase studies: first Italian nationwide survey.Eur Radiol. 2014 2; 24(2):469–83. . 24121713 10.1007/s00330-013-3031-7

[17] Zarb F, McEntee M, Rainford L. Maltese CT doses for commonly performed examinations demonstrate alignment with published DRLs across Europe. Radiat Prot Dosimetry. 2012 6;150(2):198–206. 10.1093/rpd/ncr393 . 21993803

[18] Smith AB, Dillon WP, Gould R, Wintermark M. Radiation dose-reduction strategies for neuroradiology CT protocols. Am J Neuroradiol. 2007 10;28(9):1628–32. . 17893208 10.3174/ajnr.A0814 PMC8134195

[19] McNitt-Gray MF. AAPM/RSNA physics tutorial for residents: topics in CT. Radiation dose in CT. Radiographics. 2002 Nov-Dec;22(6):1541–53. . 12432127 10.1148/rg.226025128

[20] Smith AB, Dillon WP, Lau BC, Gould R, Verdun FR, Lopez EB, et al Radiation dose reduction strategy for CT protocols: successful implementation in neuroradiology section. Radiology. 2008 5; 247(2):499–506. 10.1148/radiol.2472071054 . 18372456

[21] Huda W, Magill D, He W. CT effective dose per dose length product using ICRP 103 weighting factors. Med Phys. 2011 3; 38(3):1261–5. . 21520838 10.1118/1.3544350

[22] Shrimpton PC, Wall BF. Reference dosimetry for CT in the UK. Internation Atomic Energy Agency. 2000. Available: http://www.iaea.org/inis/collection/NCLCollectionStore/_Public/32/039/32039909.pdf.

[23] ACR–ASNR Practice Guideline for the Performance of Computed Tomography (CT) of the Brain. 2010. Available: http://www.guideline.gov/content.aspx?id=32518.

[24] Wu TH, Hung SC, Sun JY, Lin CJ, Lin CH, Chiu CF, et al How far can the radiation dose be lowered in head CT with iterative reconstruction? Analysis of imaging quality and diagnostic accuracy. Eur Radiol. 2013 Sep; 23(9):2612–21. 10.1007/s00330-013-2846-6 . 23645331

[25] Korn A, Bender B, Fenchel M, Spira D, Schabel C, Thomas C, et al Sinogram Affirmed iterative reconstruction in head CT: Improvement of objective and subjective image quality with concomitant radiation dose reduction.Eur J Radiol. 2013 9; 82(9):1431–5. 10.1016/j.ejrad . 23587902

[26] Lo¨ve A, Siemund R, Ho¨glund P, Van Westen D, Stenberg L, Petersen C, et al Hybrid iterative reconstruction algorithm in brain CT: a radiation dose reduction and image quality assessment study. Acta Radiol. 2014 3;55(2):208–17. 10.1177/0284185113494980 . 23897306

[27] Russell MT, Fink JR, Rebeles F, Kanal K, Ramos M, Anzai Y. Balancing radiation dose and image quality: clinical applications of neck volume CT. Am J Neuroradiol. 2008 4;29(4):727–31. 10.3174/ajnr.A0891 . 18223095 PMC7978204

